# Toward Transparent and Controllable Quantum Generative Models

**DOI:** 10.3390/e26110987

**Published:** 2024-11-17

**Authors:** Jinkai Tian, Wenjing Yang

**Affiliations:** 1 Intelligent Game and Decision Lab, Beijing 100071, China; 2 Department of Intelligent Data Science, College of Computer Science and Technology, National University of Defense Technology, Changsha 410073, China

**Keywords:** quantum neural networks, explainable artificial intelligence, autoencoder

## Abstract

Quantum generative models have shown promise in fields such as quantum chemistry, materials science, and optimization. However, their practical utility is hindered by a significant challenge: the lack of interpretability. In this work, we introduce *model inversion* to enhance both the interpretability and controllability of quantum generative models. Model inversion allows for tracing generated quantum states back to their latent variables, revealing the relationship between input parameters and generated outputs. We apply this method to models generating ground states for Hamiltonians, such as the transverse-field Ising model (TFIM) and generalized cluster Hamiltonians, achieving interpretability control without retraining the model. Experimental results demonstrate that our approach can accurately guide the generated quantum states across different quantum phases. This framework bridges the gap between theoretical models and practical applications by providing transparency and fine-tuning capabilities, particularly in high-stakes environments like drug discovery and material design.

## 1. Introduction

Quantum generative models [1] have shown significant potential in fields such as drug discovery and materials science [2], leveraging quantum mechanics to efficiently generate complex quantum states or data samples [3]. However, these models face unique interpretability challenges due to quantum properties like superposition and entanglement, which contribute to high entropy in the state space and complicate output interpretation.

A primary challenge is the difficulty in interpreting the relationship between input variables (e.g., Hamiltonian parameters) and output quantum states [4,5,6,7,8]. Unlike classical models, where layers and parameters can often be directly analyzed, quantum models operate within highly abstract quantum state spaces. The “black box” nature of quantum models limits our understanding of their internal operations, making it challenging to control or verify specific outcomes, especially in high-stakes applications.

This lack of interpretability has significant practical implications. In critical fields like drug discovery and materials science, understanding how quantum states are generated is vital for ensuring trustworthy results that meet stringent requirements. Without the ability to interpret the relationships between input parameters and generated quantum states, controlling these models to achieve desired outcomes becomes extremely difficult, undermining their reliability and broader adoption.

While classical generative models also face interpretability challenges, various techniques—such as visualization tools, simplified architectures, and model inversion—have been developed to enhance understanding [9,10]. However, these methods are often insufficient or inapplicable to quantum models due to their probabilistic nature and complex non-linearities, creating a critical gap in our ability to understand and control quantum generative models.

To address these challenges, we propose applying model inversion techniques to quantum generative models. Model inversion maps generate quantum states back to their latent variables, revealing the underlying relationships between inputs and outputs. This approach allows users to trace how specific quantum states are produced, offering a mechanism to control and fine-tune outputs by adjusting latent variables. Moreover, it enables the identification of specific directions in the latent space that correlate with distinct features in the generated samples, allowing for targeted interventions in the generative process.

By enhancing both interpretability and control, model inversion directly addresses the critical gap in current research. It provides a way to understand and manipulate the complex, probabilistic nature of quantum models, building trust and enabling more precise control over outputs—being especially important in applications where fine-tuning quantum states is essential.

In this study, we apply model inversion techniques to quantum generative models, focusing on generating ground states for Hamiltonians such as the transverse-field Ising model (TFIM) and generalized cluster Hamiltonians. Our approach not only enhances interpretability but also provides a pathway for controlling outputs, making quantum models more reliable and adaptable for practical applications.

By addressing interpretability and control challenges, this work contributes to bridging the gap between the theoretical promise of quantum generative models and their practical utility. With model inversion, we offer a method that allows researchers and practitioners to explore the internal workings of quantum models more transparently, providing both control and flexibility—critical elements for deploying quantum generative models in real-world applications, where precision, reliability, and interpretability are paramount.

## 2. Preliminary

### 2.1. Quantum Generative Models

Quantum generative models have emerged as powerful tools for simulating and generating quantum states across various quantum systems. These models leverage quantum computing’s inherent ability to handle complex, high-dimensional state spaces, offering a range of approaches for state generation. One such class is the Quantum Circuit Born Machine (QCBM) [11,12], which uses quantum circuits to learn and represent probabilistic distributions of quantum states. QCBMs are particularly effective at learning quantum data distributions and can be applied to quantum state preparation and quantum machine learning tasks. Another prominent class is Quantum Generative Adversarial Networks (QGANs) [13,14], which apply a competitive learning process similar to classical GANs, where a quantum generator creates states that a discriminator (classical or quantum) evaluates, allowing for high-fidelity state generation.

Each of these quantum generative models has demonstrated success in tasks such as quantum state preparation, quantum state tomography, and quantum simulations. However, despite these advancements, significant challenges remain. In particular, understanding and controlling the generated quantum states pose serious difficulties due to the complexity of quantum systems and the probabilistic nature of quantum mechanics. In this context, studying specific quantum systems, such as the transverse-field Ising model (TFIM) and generalized cluster Hamiltonian, provides a structured approach to evaluating and understanding quantum generative models, as they represent well-known quantum phase transition systems that can serve as benchmarks for state generation tasks.

### 2.2. Quantum Phase Transitions

Quantum phase transitions are phase transitions driven by quantum fluctuations, which occur at absolute zero (0 Kelvin) as a parameter in the Hamiltonian changes [15]. Two prominent models in the study of quantum phase transitions are the transverse-field Ising model (TFIM) and the generalized cluster Hamiltonian.

The Hamiltonian for the one-dimensional TFIM is given by

(1) $$H=-J\sum_{i}\sigma_{z}^{i}\sigma_{z}^{i+1}-h\sum_{i}\sigma_{x}^{i},$$

where *J* represents the interaction strength between neighboring spins, *h* denotes the transverse magnetic field strength, and $\sigma_{x}^{i}$ and $\sigma_{z}^{i}$ are the Pauli-X and Pauli-Z operators acting on the *i*-th spin, respectively. In the TFIM, quantum phase transitions occur at the critical point where the relative strength between the transverse magnetic field *h* and the spin–spin interaction *J* changes. Specifically, for the one-dimensional TFIM, the critical point is reached at J=h, leading to a quantum phase transition from an ordered phase (J>h) to a disordered phase (J<h). Experimental studies have demonstrated similar transitions between thermalized (disordered) and localized (ordered) states, such as in the work by Guo et al. [16], where entropy plays a critical role in characterizing these phases.

In contrast, the ground state of the generalized cluster Hamiltonian resides in a symmetry-protected topological (SPT) phase, which exhibits greater complexity than the TFIM. This model has been employed to evaluate the performance of quantum neural networks [17]. The generalized cluster Hamiltonian is defined as

(2) $$H=\sum_{j}\left(\sigma_{z}^{j}-J_{1}\sigma_{x}^{j}\sigma_{x}^{j+1}-J_{2}\sigma_{x}^{j-1}\sigma_{z}^{j}\sigma_{x}^{j+1}\right),$$

where $J_{1}$ and $J_{2}$ represent distinct interaction strengths. As demonstrated by [18], the phase diagram for this Hamiltonian includes four distinct phases: the symmetry-protected topological phase (I), ferromagnetic phase (II), antiferromagnetic phase (III), and trivial phase (IV).

To investigate these models, we constructed a TFIM dataset, $D=\{\left(|\psi_{i}\rangle ,y_{i}\right)\}$, consisting of 500 data points. Each data point corresponds to the ground state of a 4-qubit TFIM, where $y_{i}$ is labeled as 1 (ordered phase) or 0 (disordered phase) depending on the quantum state. Additionally, we developed a symmetry-protected topological state (SPTS) dataset with four classifications, containing 300 data points, based on the 4-qubit cluster Hamiltonian model.

## 3. Methodology

The study of quantum phase transitions highlights the intricate behavior of quantum systems as they transition between different phases, which is often controlled by parameters such as interaction strengths or external fields. Understanding and controlling these quantum transitions is crucial in various applications, from condensed matter physics to quantum computing. However, achieving precise control over the quantum states involved in these transitions remains a significant challenge due to the complex, non-linear relationships between the system’s parameters and the resulting quantum phases.

To address this challenge, our approach leverages a technique called model inversion. At its core, model inversion provides a means to trace the path from generated quantum states back to the latent variables that produced them. This approach offers a critical advantage: it not only enables a deeper understanding of how specific quantum states—such as those observed in quantum phase transitions—are generated but also provides a pathway for controlling the generation process itself. By mapping output quantum states back to their latent space, we can optimize these latent variables, allowing for precise adjustments that produce specific, desired quantum states or properties. This is particularly useful in navigating the complex landscape of quantum phases, where small changes in parameters can lead to significant shifts in the system’s behavior.

Unlike classical models, quantum systems exhibit unique properties such as superposition and entanglement, where small changes in latent variables can lead to significant and non-trivial transformations in the output quantum state. By applying model inversion, we gain a level of precision that allows us to control specific attributes of the quantum states being generated, such as energy levels or symmetry properties. The method also enables the identification of specific latent directions associated with distinct quantum phenomena, such as different quantum phases, offering new avenues for practical manipulation and refinement of quantum outputs.

### 3.1. Model Design

In this section, we present the design of a quantum generative model aimed at generating the ground state of generalized cluster Hamiltonians (SPTS) while offering interpretability control through model inversion techniques. Currently, training a variational quantum eigensolver [19] is limited to obtaining the ground state of a specific Hamiltonian. The objective of this model design is to handle a class of Hamiltonians by encoding the parameters $J_{1}$ and $J_{2}$ of the generalized cluster Hamiltonian using rotation gates, thereby directly solving for the ground state of the Hamiltonian. Experimental results demonstrate that this approach achieves a fidelity exceeding 80%. Further improvements to the generator can be achieved by employing meta-learning methods, enabling a single model to generate ground states for a broader class of Hamiltonians [20].

The motivation behind this design is rooted in the challenges posed by quantum phase transitions, where the relationship between Hamiltonian parameters and quantum phases is often highly non-linear. Model inversion provides a critical mechanism for tracing this relationship, allowing us to identify latent variables and adjust them to generate desired quantum states. By offering this level of control, the quantum generative model becomes a powerful tool for studying complex quantum systems, such as the TFIM and generalized cluster Hamiltonians. Additionally, interpretability control enables researchers to fine-tune quantum models without redesigning the entire architecture, which is particularly advantageous for high-stakes quantum computing applications.

**Figure 1:**
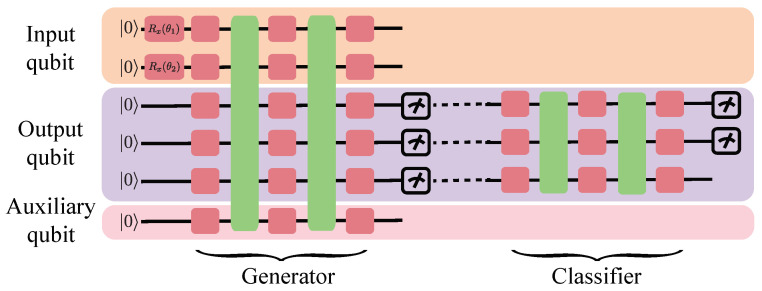
Quantum circuit diagram of the quantum generative model for interpretability control. The red boxes represent parameterized rotation gates: the two marked red boxes use Pauli-X rotation gates, while all other unmarked red boxes use Pauli-Y rotation gates. The green boxes denote entanglement layers composed of CNOT gates applied in sequence according to qubit order.

As depicted in Figure 1, the model comprises components: a generator and a classifier. The generator takes Hamiltonian information or random vectors as input and generates quantum states. The classifier is employed to guide the direction of model inversion.

The quantum bits in the system are divided into three categories: input qubits (i), output qubits (o), and auxiliary qubits (a), with $n_{i}$, $n_{o}$, and $n_{a}$ qubits assigned to each, respectively. The input qubits are used to encode the input data via rotation gates, the output qubits are responsible for producing the reconstructed quantum state in the generator, and the auxiliary qubits are introduced to expand the model’s operational space, thus enhancing its expressive power. In the context of the SPTS problem, we directly align the latent space representation with $J_{1}$ and $J_{2}$. Consequently, after training, the generator is capable of mapping $J_{1}$ and $J_{2}$ into the ground state of the corresponding generalized cluster Hamiltonian.

The initial state of the generator is ${|0\rangle }_{i,o,a}={|0\rangle }_{i}⊗{|0\rangle }_{o}⊗{|0\rangle }_{a}$, representing a state of minimum entropy. The rotation gate encoding is applied to encode $J_{1}$ and $J_{2}$ into the generator, increasing the system’s entropy as the superposition of states becomes more complex. This process is carried out by the quantum circuit $V{\left(\theta \right)}^{\prime }$. The main structure of the generator is as follows: $V\left(\theta \right)=V{\left(\theta \right)}^{\prime }\left(R_{x}\left(\theta_{1}\right)⊗R_{x}\left(\theta_{2}\right)⊗I\right)$. The inputs $J_{1}$ and $J_{2}$ of the generator take values in (−∞,∞), while the parameters accepted by the rotation gates are periodic, i.e., $R_{z}\left(\theta +2\pi \right)=R_{z}\left(\theta \right)$. Therefore, a sigmoid function is first applied to map these values to the range (−π,π):

(3) $$\begin{array}{cc} \theta_{1} & =(2\times \sigma \left(J_{1}\right)-1)\times \pi \end{array}$$

(4) $$\begin{array}{cc} \theta_{2} & =(2\times \sigma \left(J_{2}\right)-1)\times \pi \end{array}$$

The output of the generator is the reconstructed ground state of the generalized cluster Hamiltonian:

(5) $$\rho_{o}={\mathrm{Tr}}_{i,a}\left[V\left(\theta \right){|0\rangle }_{i,o,a}{\langle 0|}_{i,o,a}V{\left(\theta \right)}^{†}\right]$$

Here, ${\mathrm{Tr}}_{i,a}$ represents the partial trace over the input and auxiliary qubits. $\rho_{o}$ is the reconstructed quantum state, which is stored in the output qubits.

The classifier is implemented using a hardware-efficient ansatz W(θ), which takes the quantum state output from the generator as input and produces a probability distribution $p=(p_{1},\ldots ,p_{n})$. This distribution is obtained by measuring the probability of the |0〉 state on the input qubits or by measuring the computational basis states of the first few qubits. During model training, the generator is first trained to reconstruct the ground state. Through supervised learning of the latent space variables, the generator can achieve the conversion between the ground state of the generalized cluster Hamiltonian and the Hamiltonian parameters $J_{1}$ and $J_{2}$.

For classifier training, the cross-entropy loss function is used. When control over the generated sample is required to shift it in a specific direction, the gradient of the model’s loss function with respect to the inputs $J_{1},J_{2}$ is calculated. Perturbations are applied to the latent space variables based on the gradient direction, thus providing control over the quantum generative model.

In this study, the interpretability control experiment was conducted in a two-dimensional latent space for a binary classification task. For multi-classification problems or higher-dimensional latent spaces, the model can be appropriately adjusted. The classification probabilities are computed by measuring the first qubit of the classifier, which is represented as $p=(p_{0},p_{1})$:

(6) $$\begin{array}{cc} p_{0} & =\mathrm{tr}[W\left(\theta \right)\rho_{o}W^{†}\left(\theta \right)\left(|0\rangle \langle 0|⊗I⊗\cdots ⊗I\right)], \end{array}$$

(7) $$\begin{array}{cc} p_{1} & =\mathrm{tr}[W\left(\theta \right)\rho_{o}W^{†}\left(\theta \right)\left(|1\rangle \langle 1|⊗I⊗\cdots ⊗I\right)] \end{array}$$

For interpretability control, if we aim to bias the generated sample toward the first class, the loss function is defined as

(8) $$L\left(p,q\right)=-\sum_{i=0}^{1}p\left(i\right)\log q\left(i\right)$$

where q=(1,0) represents the desired classification probability distribution. At this point, the update direction of the latent space vector is $\left(\frac{\partial L}{\partial J_{1}},\frac{\partial L}{\partial J_{2}}\right)$, and by adjusting the latent space variables along this direction, interpretability control of the generated sample’s classification can be achieved.

#### Data Re-Uploading Strategy

Data re-uploading is a technique that enhances the expressiveness and flexibility of quantum models by iteratively encoding input data multiple times throughout the quantum circuit [21]. In this process, the input data (such as Hamiltonian parameters) are applied to the quantum state at various points in the circuit, often in conjunction with parameterized rotation gates and entanglement layers. This allows the model to encode more complex functions and relationships, which is particularly useful for capturing the intricate patterns present in quantum datasets. By reintroducing the input data at multiple stages, the model can leverage the quantum state’s superposition and entanglement properties more effectively, leading to improved representation capabilities and better control over the generated outputs.

In our implementation, we applied data re-uploading by repeating the encoding of Hamiltonian parameters $J_{1}$ and $J_{2}$ within each layer of the quantum circuit. The parameters are first encoded using rotation gates, and as the data progresses through subsequent layers, these parameters are re-encoded to reinforce their influence on the quantum state evolution. This iterative approach enables the model to fine-tune the quantum state representation more precisely, achieving higher fidelity in generating quantum states that correspond to various Hamiltonian configurations. Additionally, the number of data re-uploading cycles directly impacts the expressiveness of the model: increasing the cycles allows for deeper exploration of the parameter space but may also require more computational resources and training time. The effectiveness of the data re-uploading strategy is validated in our subsequent experiments, where we observe that models employing this technique achieve higher classification accuracy and improved interpretability control compared to those without data re-uploading.

### 3.2. Model Inversion

In the context of a quantum state generated by a quantum generative model, the goal of model inversion is to recover the corresponding latent variables from the latent space of the model. Unlike traditional model evaluation techniques that focus solely on performance metrics, our model inversion framework provides a novel mechanism to understand the generation of quantum states by tracing the latent variables back to the generated output. This enables researchers to gain insights into the quantum generative process and provides a way to control the quantum states generated—an advancement not previously explored in quantum machine learning research. By adjusting these latent variables, specific modifications to the quantum state can be made. This method enables the direct use of pre-trained quantum generative models without requiring re-design or re-training for interpretability.

**Figure 2:**
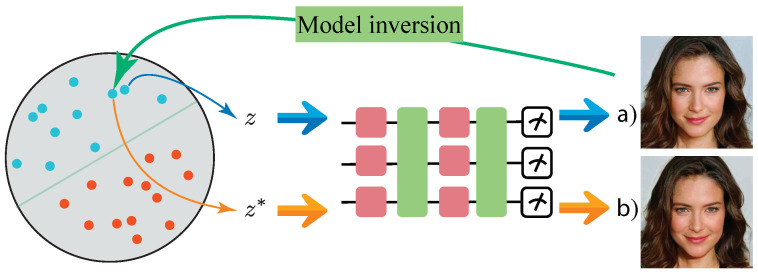
Example of model inversion method applied to quantum states. (**a**) A quantum state is generated by inputting latent variables *z* into the quantum generative model; (**b**) The resulting quantum state obtained when the inverted latent variables $z^{*}$ are input into the model, illustrating the approximation differences.

As illustrated in Figure 2, the model inversion process consists of several steps. The significance of this approach lies in its practical applications. In fields such as quantum chemistry, where precise quantum state manipulation is critical, model inversion enables the generation of ground states with specific quantum properties. This method allows for targeted interventions, guiding the quantum generative model toward desired outcomes, which is essential for tasks like simulating material properties or chemical reactions. In Figure 2a, a quantum state is generated by inputting latent variables *z* into the quantum generative model. Through model inversion, the latent variables $z^{*}$ are obtained. In most cases, $z^{*}$ is not exactly equal to *z*, as some approximation error is typically present due to the complexity of the inversion process. When $z^{*}$ was input into the generative model, the resulting quantum state, as seen in Figure 2b, usually showed slight differences from the original quantum state due to these approximations.

The mathematical formulation of model inversion can be described as follows. A quantum generative model learns a mapping G:Z→X, where Z represents the latent space, and X is the space of generated quantum states. When $z_{1},z_{2}\in Z$ are close in the latent space, the corresponding quantum states $|x_{1}\rangle ,|x_{2}\rangle \in X$ are generally very similar. The objective of model inversion is to map a quantum state |x〉 back to its latent space representation $z^{*}$. From the quantum state space perspective, this entails finding a state $|x^{*}\rangle$, which can be synthesized by a well-trained generator *G* and is as close as possible to the real quantum state |x〉. The inversion problem can be defined as

(9) $$z^{*}=\underset{z}{\arg\min}\;L\left(G\left(z\right),|x\rangle \right)$$

where L(·) is a distance metric in either the quantum state space (quantum relative entropy and quantum state fidelity) or feature space (norms such as the Manhattan norm $\ell_{1}$ or the Euclidean norm $\ell_{2}$). Utilizing entropy-based metrics allows us to quantify the difference between probability distributions of quantum states, providing a more informative inversion process.

In quantum generative models, the probabilistic nature of quantum mechanics plays a central role. Quantum states generated by these models encode probability distributions over measurement outcomes, where the probability of observing a specific outcome is determined by the quantum state’s amplitude. To ensure that our method aligns with this probabilistic framework, we used quantum relative entropy and quantum state fidelity. Quantum relative entropy quantifies the divergence between the probability distributions of two quantum states, while fidelity measures the similarity between states. These probabilistic measures were integrated into our loss function L(G(z),|x〉), ensuring that the inversion process respects the probabilistic characteristics of the generated quantum states.

### 3.3. Analysis of Internal Representations

Instead of focusing solely on exploring the latent variable space, an alternative approach for interpreting the workings of quantum generative models is to analyze their internal quantum representations. This method delves deeper into the internal quantum states generated by the model, in contrast to model inversion, which focuses on the input–output mapping. Model dissection aims to identify interpretable units that are closely associated with certain physical properties or phases of the quantum state.

Let $z\in R^{\left|z\right|}$ represent the latent vector sampled from a low-dimensional distribution and encoded onto the quantum model using data encoding, which is formally expressed as

(10) $$|z\rangle =U_{z}|0\rangle$$

Let |x〉=G(|z〉) represent its corresponding generated quantum state. We focus on the quantum state $|r\rangle =U_{1}|z\rangle$ at an intermediate layer of the generator *G*, which we refer to as the internal representation and represent it as

(11) $$|x\rangle =G\left(|r\rangle \right)=U_{2}|r\rangle =U_{2}U_{1}|z\rangle$$

Define the set of quantum properties or phases to be analyzed as C. Since |r〉 contains all the information necessary to generate the quantum state |x〉, it also carries the information needed to infer any high-level quantum property *c* from the state. The key question is not whether information about *c* is present in |r〉, but rather how this information is encoded. More specifically, for any high-level quantum property c∈C, we seek to understand whether |r〉 represents *c* in a well-defined manner. This could involve computing the gradients of the probability amplitudes of |r〉 using model inversion methods, thereby enabling control and adjustment of the output by manipulating these amplitudes.

### 3.4. Controllability for Interpretability

To control and adjust the properties of generated quantum states, specific operations must be applied to the latent vector $z^{*}$ obtained through model inversion. The control of the quantum generative model’s output generally depends on two factors: first, identifying semantically meaningful directions in the latent variable space that correspond to specific quantum phases or properties, and second, performing appropriate operations in this space to modify the generated quantum states accordingly.

For example, to fine-tune generated quantum states in the context of a quantum phase transition, perturbations can be introduced to the latent variable $z^{*}$ obtained through model inversion. This is achieved by inputting the quantum generative model’s output into a quantum phase classifier *D* and calculating the direction of perturbation $\vec{a}$. The process is described by the following equation:

(12) $$\vec{a}=\nabla_{z^{*}}L\left(D\left(G\left(z^{*}\right)\right),y\right),$$

where $D(G\left(z^{*}\right))$ represents the classification result obtained from the quantum phase classifier, and *y* denotes the target phase or property. The difference between these is computed using the loss function L. Here, *y* represents the desired target phase for the generated quantum state.

**Figure 3:**
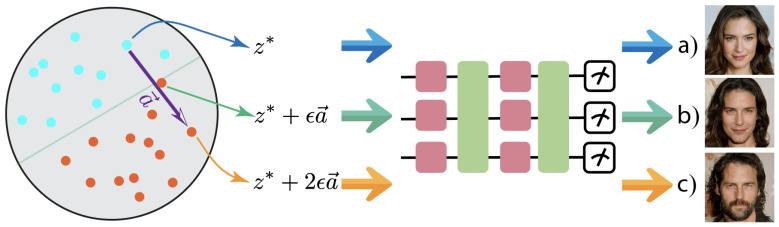
Example of controllability applied to quantum phase transitions. To visualize the quantum state, we use an illustrative analogy. (**a**) Initial quantum state; (**b**) Result after adjustment with a step size ϵ; (**c**) Result after adjustment with a step size 2ϵ.

As demonstrated in Figure 3, this method can be used to transition a quantum state between different phases, such as from a high-entropy disordered phase to a low-entropy ordered phase, by adjusting the latent variables. The larger the step size, the more the quantum state changes its phase characteristics, offering precise control over the quantum generative model. This approach provides a powerful tool for exploring quantum phase transitions and controlling quantum states in a generative model, without requiring additional costly training.

In our implementation, we used the gradient descent method, specifically the Adam optimizer [22], to minimize Equation (12). This approach is effective for the relatively low-dimensional latent space in our experiments. However, for higher-dimensional latent spaces, more advanced techniques, such as gradient clipping or adaptive learning rate strategies, may be necessary to maintain stable convergence and manage the increased complexity.

### 3.5. Significance of Model Inversion for Quantum Generative Models

The introduction of model inversion in quantum generative models provides a dual benefit of interpretability and control. By mapping generated quantum states back to their latent variables, this method allows for a deeper understanding of the generative process and offers a unique capability to fine-tune the output. Unlike conventional tuning methods, which adjust model parameters to improve accuracy or performance, model inversion enables direct manipulation of quantum state properties, making it invaluable for high-stakes applications like quantum chemistry or quantum phase transition simulations.

Furthermore, the flexibility of this approach allows it to be applied to more complex quantum systems where inputs are not easily described by Hamiltonian parameters or where traditional methods fail to provide insight into the underlying quantum phenomena. This makes our contribution a significant step forward in bridging the gap between theoretical advancements in quantum generative models and their practical applicability in real-world quantum systems.

## 4. Experiments

### 4.1. Experimental Setup

We conducted experiments using datasets generated from the transverse-field Ising model (TFIM) and the generalized cluster Hamiltonian model, each with 2000 samples. Each sample consists of the Hamiltonian parameters (e.g., $J_{1}$ and $J_{2}$ for the SPTS dataset), the corresponding ground state quantum wavefunction, and classification labels indicating the phase.

We considered a 4-qubit TFIM Hamiltonian with varying transverse field strength *h* and interaction strength *J*. The ground states were computed numerically for different values of *h* and *J*, covering both ordered and disordered phases. Each ground state was labeled as either ordered (label 1) or disordered (label 0) based on the phase. For the generalized cluster Hamiltonian, we generated ground states for various combinations of $J_{1}$ and $J_{2}$, covering the four distinct phases (I: symmetry-protected topological phase, II: ferromagnetic phase, III: antiferromagnetic phase, and IV: trivial phase), as identified in [18]. The ground states were computed for a 4-qubit system and labeled accordingly.

The generator is a parameterized quantum circuit designed to map latent variables (Hamiltonian parameters $J_{1}$ and $J_{2}$) to quantum states approximating the ground states of the Hamiltonians. The generator circuit comprises multiple layers, each consisting of parameterized single-qubit rotation gates ($R_{x}$ and $R_{y}$) and entangling gates (CNOT gates). The latent variables are encoded into the circuit via rotation gates. The classifier is another parameterized quantum circuit that takes the generated quantum states as input and outputs probabilities corresponding to different phases. It uses a hardware-efficient ansatz with layers of rotation gates and entangling gates. Key configuration details are summarized in Table 1.

The models were implemented using the Pennylane quantum computing (version 0.39, created by Xanadu, Toronto, ON, Canada) framework [23]. The loss function for the generator includes a fidelity term measuring the similarity between the generated state and the true ground state. The Adam optimizer was used for parameter updates, with hyperparameters $\beta_{1}=0.9$, $\beta_{2}=0.999$, and ϵ= $1\times 10^{-8}$. Training was performed for 10 epochs, and model parameters were updated to minimize the loss function. To assess the impact of data re-uploading, we experimented with different configurations (1 × 30 layers, 3 × 10 layers, 5 × 6 layers), where the total number of layers was kept constant, but the data re-uploading frequency was varied.

**Figure 4:**
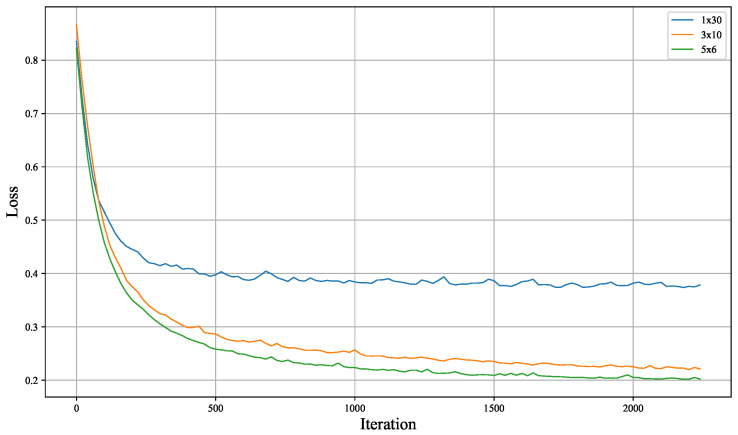
Training loss curve of the quantum generative model on the TFIM dataset. The legend represents (number of data re-uploadings × number of layers after each re-uploading).

**Figure 5:**
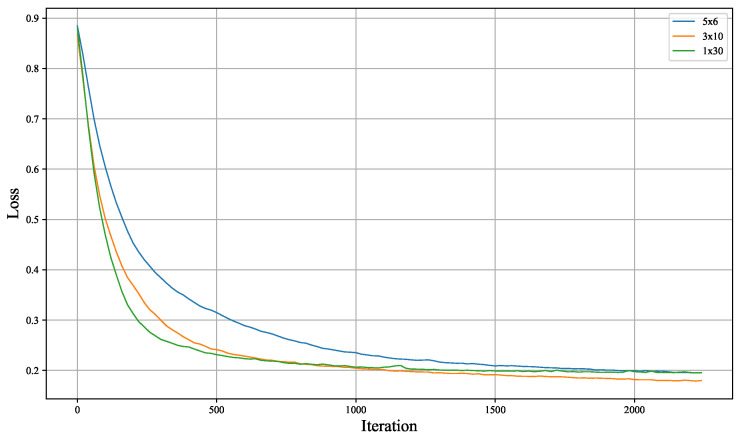
Training loss curve of the quantum generative model on the SPTS dataset.

To assess the effect of data re-uploading on the generator, various re-uploading strategies were applied to the TFIM and SPTS datasets. The training dynamics are shown in Figure 4 and Figure 5. Key observations from the training process are as follows:

- Both the TFIM and SPTS datasets achieved fidelity levels exceeding 80%, demonstrating successful interpretability control of the generated samples.
- The final layer of the model should exclusively involve single-qubit rotation gates, avoiding two-qubit entangling gates. Including entangling gates in the final layer significantly affects convergence speed, as two-qubit gates introduce unnecessary complexity if entanglement is not required. In contrast, single-qubit rotation gates adjust individual qubits and, if no adjustment is needed, the rotation parameters are set to zero during training. The presence of entangling gates, however, demands additional training steps to learn inverse unitary operations for unnecessary entanglement introduced in earlier layers, which increases training difficulty.
- Data re-uploading, while increasing the complexity of training, can lead to a lower final training loss if sufficient training is performed, even when the same number of layers is used.

### 4.2. Experimental Validation of Interpretability Control

**Figure 6:**
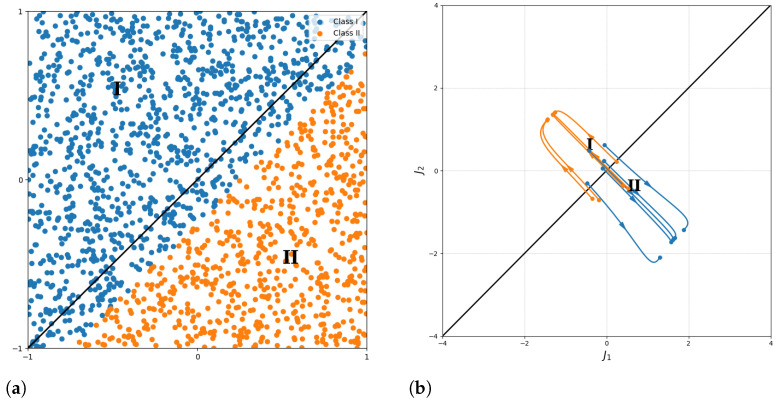
Interpretability control on TFIM data of the 1 × 30 generator. (**a**) Classification of different configurations of TFIM Hamiltonian ground states by the trained classifier, where blue and orange represent data points classified as category one and category two, respectively. (**b**) Interpretability control results for generated samples in latent space, where blue and orange indicate data points trajectory controlled toward category one and category two, respectively.

Our experiments demonstrate not just the evaluation of model performance but also the ability to control the generated quantum states through latent variable manipulation. This goes beyond typical hyperparameter tuning by offering a method to adjust the generative process itself, leading to the creation of targeted quantum states based on desired properties such as phase transitions. We first analyzed the classification performance and interpretability control results on the relatively simple TFIM dataset, as shown in Figure 6. Five samples were randomly selected from each class. The classifier’s gradient was used to guide the adjustment of disordered phase (I) samples toward the ordered phase (II) and vice versa. Figure 6a shows the classification of different configurations of TFIM Hamiltonian ground states by the trained classifier. While there were classification errors near the decision boundary, increasing the number of quantum neural network layers and re-uploading iterations managed to improve the classification accuracy. Figure 6b presents the interpretability control results for generated samples in latent space, where all samples from both classes were successfully guided toward the target classifications.

Each trajectory in Figure 6 represents the path generated through interpretability control. We set the step size to 0.5 and ran the process for 30 steps to obtain each curve. The quality of the perturbation depends on the sample’s initial position relative to the decision boundary; samples closer to the boundary generally require smaller perturbations to reach the target classification.

**Figure 7:**
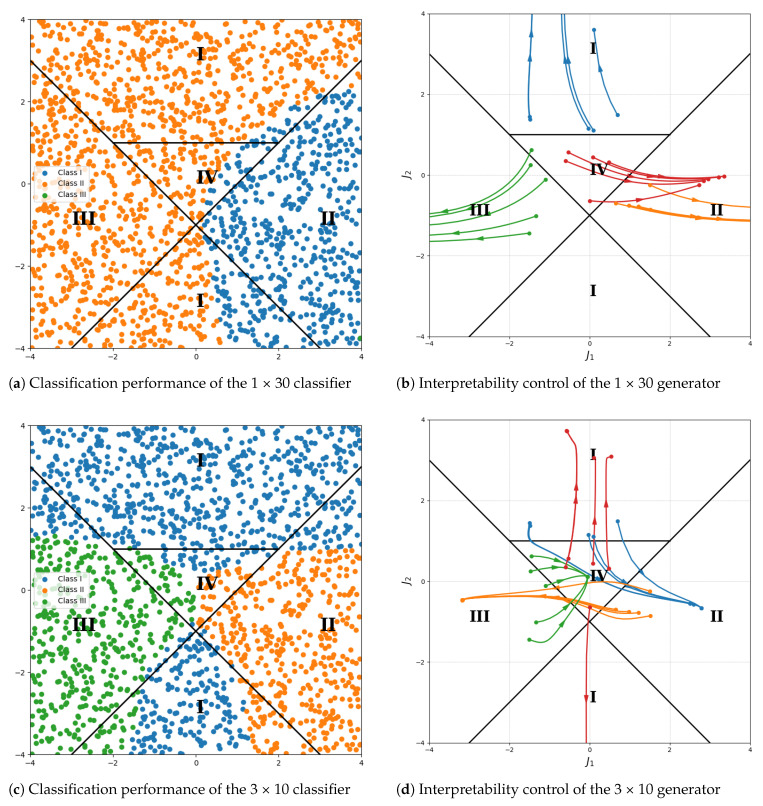

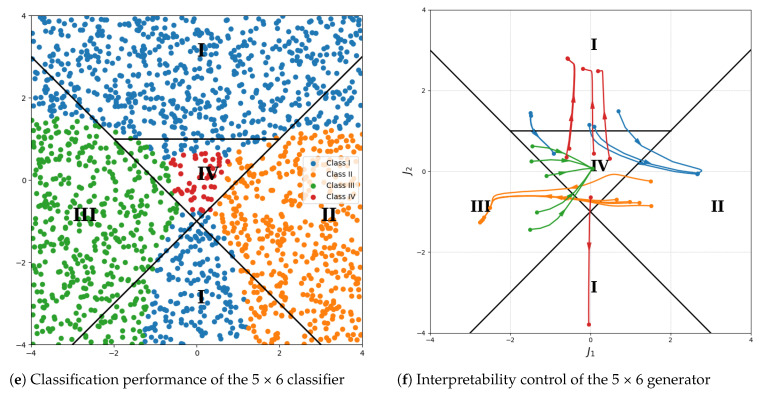
Interpretability control of quantum generative models by altering the data re-uploading strategy while keeping the number of neural network layers unchanged. In subfigures (**a**,**c**,**e**), blue, orange, green, and red represent data points classified into categories one, two, three, and four, respectively. In subfigures (**b**,**d**,**f**), red, blue, orange, and green indicate the trajectories of data points being controlled toward categories one, two, three, and four, respectively.

For the more complex SPTS dataset, we analyzed different configurations of the data re-uploading strategy, as shown in Figure 7. Four categories—symmetry-protected topological phase (I), ferromagnetic phase (II), antiferromagnetic phase (III), and trivial phase (IV)—were used, with five randomly selected samples from each category. Interpretability control was applied to adjust samples from I to II, II to III, III to IV, and IV to I.

In the SPTS experiments, as shown in Figure 7a,c,e, we found that data re-uploading helped manage complex classification tasks and addressed the multi-classification problem more effectively. Figure 7a indicates that without data re-uploading, the classifier could only divide the samples into two classes. Figure 7c shows that with the 3 × 10 configuration, three classes of samples were identifiable, while Figure 7e demonstrates that the 5 × 6 configuration allowed the classifier to distinguish the intermediate IV phase.

Further analysis of interpretability control with the three classifiers is shown in Figure 7b,d,f. In Figure 7b, the latent space vector changes reveal that the model did not learn meaningful control information, as all samples moved outward from the origin. However, in Figure 7d,f, where data re-uploading was applied, the classifier provided effective guidance, directing the latent space representations toward the target regions: I→II,II→III,III→IV,IV→I.

## 5. Conclusions

In summary, our work presents a solution to one of the most pressing challenges in the field of quantum generative models: interpretability. Through the development and application of model inversion techniques, we provide a framework that enhances both the understanding and control of quantum state generation. This method bridges the gap between the theoretical promise of quantum generative models and their practical applicability, especially in high-stakes fields where precise control is paramount.

By analyzing the latent and intermediate state spaces, we gain insights into how quantum generative models learn and generate features. The high-dimensional latent space encodes specific attributes of generated quantum states, and understanding how movements in this space affect the output helps clarify the model’s learning process. Furthermore, model inversion allows for the generation and fine-tuning of customized samples based on specific attributes, enabling users to control the output to meet particular requirements. This flexibility extends to tasks like style transfer or adjusting quantum state phases, without the need for model re-training, making the approach applicable in fields such as design, art, and media production.

While our experiments focus on a relatively simple and typical scenario, where the inputs are set as Hamiltonian parameters and the phase diagram is well known, the proposed model inversion technique is not limited to such cases. This algorithm is applicable to a wider range of quantum systems, including those where the inputs cannot be easily described by Hamiltonian parameters or where the phase diagram is unknown. It is also robust for more complex systems with irreversible generative processes, where noise or quantum coherence complicates the inversion process.

Moreover, the interpretability control framework extends to quantum tasks with non-linear or probabilistic generative processes, such as quantum optimization or systems with unknown properties. In scenarios where explicit parameters are unavailable or quantum states undergo irreversible transitions, our algorithm still enables control and fine-tuning, showcasing its versatility across diverse quantum systems.

Looking ahead, we recognize that an alternative approach to enhancing interpretability is to analyze the internal quantum representations within the generative models. Instead of focusing solely on exploring the latent variable space, examining the internal quantum states generated by the model can provide deeper insights into how the model processes information. This method involves identifying interpretable units or intermediate quantum states that are closely associated with certain physical properties or phases of the quantum system.

For instance, by focusing on the quantum state at an intermediate layer of the generator—referred to as the internal representation—we can investigate how high-level quantum properties are encoded within the model. The key question is not whether information about a specific property is present in the internal state, but rather how this information is encoded. Understanding this encoding could involve computing the gradients of the probability amplitudes of the internal state using model inversion methods, thereby enabling control and adjustment of the output by manipulating these amplitudes.

However, examining intermediate quantum states presents certain limitations in the context of interpretability control. Specifically, while directly modifying artificially constructed input states is feasible, editing or manipulating the quantum states produced by intermediate layers of a QNN is challenging due to measurement constraints, quantum decoherence, and the complexity of state reconstruction. Intermediate quantum states within a QNN cannot be directly measured without disrupting the quantum computation, owing to the no-cloning theorem and the collapse of the wavefunction upon measurement. Additionally, reconstructing these states would require quantum state tomography, which is resource-intensive and impractical for systems with more than a few qubits.

Given these limitations, focusing on input–output relationships through model inversion offers a more practical and effective approach for enhancing interpretability and control in quantum generative models at this stage. Nevertheless, we believe that exploring internal quantum representations remains a promising avenue for future research. Developing methods to infer the properties of intermediate states without direct measurement or designing architectures that facilitate better interpretability of internal processes could overcome current challenges and further enhance the capabilities of quantum generative models.

As quantum computing continues to evolve, our approach to interpretability can serve as a foundation for future research, potentially extending to other areas such as quantum optimization, quantum discriminative machine learning, and beyond. The ability to both interpret and control these models will open up new possibilities, driving advancements in quantum technologies and their applications.

## Figures and Tables

**Table 1:** Configuration of the generator for interpretability control experiments.

Parameter	Value
Number of input qubits	2
Number of output qubits	3
Number of auxiliary qubits	1
Total number of qubits	6
Number of layers	30
Data re-uploading cycles	1 × 30, 3 × 10, 5 × 6
Encoding method	Rotation gate encoding
Optimizer	Adam
Learning rate	0.001
Batch size	8
Epochs	10

## Data Availability

The original data and codes presented in the study are openly available at https://github.com/kkrusher/quantum-generative-explanation (accessed on 12 November 2024).

## References

[1] Tian J., Sun X., Du Y., Zhao S., Liu Q., Zhang K., Yi W., Huang W., Wang C., Wu X. (2023). Recent Advances for Quantum Neural Networks in Generative Learning. IEEE Trans. Pattern Anal. Mach. Intell..

[2] Cao Y., Romero J., Olson J.P., Degroote M., Johnson P.D., Kieferová M., Kivlichan I.D., Menke T., Peropadre B., Sawaya N.P. (2019). Quantum Chemistry in the Age of Quantum Computing. Chem. Rev..

[3] Wang X., Du Y., Luo Y., Tao D. (2021). Towards Understanding the Power of Quantum Kernels in the NISQ Era. Quantum.

[4] Burge I., Barbeau M., Garcia-Alfaro J. (2023). A Quantum Algorithm for Shapley Value Estimation. arXiv.

[5] Heese R., Gerlach T., Mücke S., Müller S., Jakobs M., Piatkowski N. (2023). Explaining Quantum Circuits with Shapley Values: Towards Explainable Quantum Machine Learning. arXiv.

[6] Mercaldo F., Ciaramella G., Iadarola G., Storto M., Martinelli F., Santone A. (2022). Towards Explainable Quantum Machine Learning for Mobile Malware Detection and Classification. Appl. Sci..

[7] Pira L., Ferrie C. (2024). On the Interpretability of Quantum Neural Networks. arXiv.

[8] Steinmüller P., Schulz T., Graf F., Herr D. (2022). eXplainable AI for Quantum Machine Learning. arXiv.

[9] Creswell A., White T., Dumoulin V., Arulkumaran K., Sengupta B., Bharath A.A. (2018). Generative Adversarial Networks: An Overview. IEEE Signal Process. Mag..

[10] Fredrikson M., Jha S., Ristenpart T. Model Inversion Attacks That Exploit Confidence Information and Basic Countermeasures. Proceedings of the 22nd ACM SIGSAC Conference on Computer and Communications Security.

[11] Benedetti M., Garcia-Pintos D., Perdomo O., Leyton-Ortega V., Nam Y., Perdomo-Ortiz A. (2019). A Generative Modeling Approach for Benchmarking and Training Shallow Quantum Circuits. NPJ Quantum Inf..

[12] Coyle B., Mills D., Danos V., Kashefi E. (2020). The Born Supremacy: Quantum Advantage and Training of an Ising Born Machine. npj Quantum Inf..

[13] Dallaire-Demers P.L., Killoran N. (2018). Quantum Generative Adversarial Networks. Phys. Rev. A.

[14] Huang K., Wang Z.A., Song C., Xu K., Li H., Wang Z., Guo Q., Song Z., Liu Z.B., Zheng D. (2021). Quantum Generative Adversarial Networks with Multiple Superconducting Qubits. NPJ Quantum Inf..

[15] Vojta M. (2003). Quantum Phase Transitions. Rep. Prog. Phys..

[16] Guo Q., Cheng C., Sun Z.H., Song Z., Li H., Wang Z., Ren W., Dong H., Zheng D., Zhang Y.R. (2021). Observation of Energy-Resolved Many-Body Localization. Nat. Phys..

[17] Caro M.C., Huang H.Y., Cerezo M., Sharma K., Sornborger A., Cincio L., Coles P.J. (2022). Generalization in Quantum Machine Learning from Few Training Data. Nat. Commun..

[18] Verresen R., Moessner R., Pollmann F. (2017). One-Dimensional Symmetry Protected Topological Phases and Their Transitions. Phys. Rev. B.

[19] Peruzzo A., McClean J., Shadbolt P., Yung M.H., Zhou X.Q., Love P.J., Aspuru-Guzik A., O’Brien J.L. (2014). A Variational Eigenvalue Solver on a Photonic Quantum Processor. Nat. Commun..

[20] Cervera-Lierta A., Kottmann J.S., Aspuru-Guzik A. (2021). Meta-Variational Quantum Eigensolver: Learning Energy Profiles of Parameterized Hamiltonians for Quantum Simulation. PRX Quantum.

[21] Pérez-Salinas A., Cervera-Lierta A., Gil-Fuster E., Latorre J.I. (2020). Data Re-Uploading for a Universal Quantum Classifier. Quantum.

[22] Kingma D.P., Ba J. (2017). Adam: A Method for Stochastic Optimization. arXiv.

[23] Bergholm V., Izaac J., Schuld M., Gogolin C., Ahmed S., Ajith V., Alam M.S., Alonso-Linaje G., AkashNarayanan B., Asadi A. (2022). PennyLane: Automatic Differentiation of Hybrid Quantum-Classical Computations. arXiv.

